# Down-Regulation of MiR-127 Facilitates Hepatocyte Proliferation during Rat Liver Regeneration

**DOI:** 10.1371/journal.pone.0039151

**Published:** 2012-06-15

**Authors:** Chuanyong Pan, Huan Chen, Lianghua Wang, Shengsheng Yang, Hailong Fu, Yongxia Zheng, Mingyong Miao, Binghua Jiao

**Affiliations:** 1 Department of Biochemistry and Molecular Biology, Second Military Medical University, Shanghai, China; 2 Department of Anaesthesiology, Changzheng Hospital, Second Military Medical University, Shanghai, China; Centro de Investigación en Medicina Aplicada (CIMA), Spain

## Abstract

Liver regeneration (LR) after partial hepatectomy (PH) involves the proliferation and apoptosis of hepatocytes, and microRNAs have been shown to post-transcriptionally regulate genes involved in the regulation of these processes. To explore the role of miR-127 during LR, the expression patterns of miR-127 and its related proteins were investigated. MiR-127 was introduced into a rat liver cell line to examine its effects on the potential target genes *Bcl6* and *Setd8*, and functional studies were undertaken. We discovered that *miR-127* was down-regulated and inversely correlated with the expression of Bcl6 and Setd8 at 24 hours after PH, a time at which hypermethylation of the promoter region of the miR-127 gene was detected. Furthermore, in BRL-3A rat liver cells, we observed that overexpression of miR-127 significantly suppressed cell growth and directly inhibited the expression of Bcl6 and Setd8. The results suggest that down-regulation of miR-127 may be due to the rapid methylation of its promoter during the first 24 h after PH, and this event facilitates hepatocyte proliferation by releasing Bcl6 and Setd8. These findings support a miRNA-mediated negative regulation pattern in LR and implicate an anti-proliferative role for miR-127 in liver cells.

## Introduction

As a developed organ with tremendous potential for regeneration, the rat liver can grow back to its original mass and function within 7–10 days after a 70% partial hepatectomy (PH). Normally quiescent adult hepatocytes quickly enter and progress through the cell cycle in a highly synchronized manner, and these processes are automatically terminated after the liver mass is restored [1]. Despite a significant studies that have addressed the regulatory network of liver regeneration (LR) induced by PH (see [2], [3], [4] for review), many aspects of this regulatory mechanism are incompletely understood. In particular, the cellular and molecular events that regulate the early stage of LR still must be defined, and the identification of unknown markers (such as noncoding RNAs) may lend insight into the post-PH regeneration process.

MicroRNAs (miRNAs) are a group of endogenous, small, noncoding RNAs that have been shown to post-transcriptionally regulate genes by binding to the 3′-untranslated regions (UTRs) of messenger RNA (mRNA). MiRNAs have been reported to modulate a variety of biological processes, including cell differentiation and proliferation, development, metabolic regulation and apoptosis [5], [6], [7]. Recently, various studies have suggested that miRNAs may play an important part in LR. For example, up-regulated miR-21 was reported to be involved in regulating the proliferative phase of LR by targeting Pellino-1 (*Peli1*) [8] and B-cell translocation gene 2 (*Btg2*) [9], Down-regulated miR-378 has a regulatory role by inhibiting DNA-synthesis-promoting gene ornithine decarboxylase (*Odc1*) during the early phase of LR [9]. MiR-34a is up-regulated and contributes to the suppression of hepatocyte proliferation by inhibiting inhibin βB (*Inhbb*) and *Met* as described in our previous studies [10]. Especially, it has been reported that 70% of miRNAs were down-regulated 24 hours (h) after PH by a genomewide miRNA microarray study [11]. Considering the general down-regulation of miRNAs in human cancers [12], [13] and that pre-stage of hepatocellular carcinoma (HCC) shares some resemblance with LR by a transcriptional microarray analysis [14], we hypothesised that the down-regulation of miRNAs in the early stage may have critical roles in the synchronized process of LR, although these miRNAs must still be illustrated.

In a preliminary study we found that miR-127 was down-regulated 3 h after PH using a microarray analysis, and was especially prominent at 24 h by a quantitative real-time PCR (qRT-PCR) analysis. MiR-127 is typically expressed as part of a miRNA cluster in normal cells, but not in cancer cells, and this miRNA has a tumor-repressive function by targeting the pro-oncogene B-cell lymphoma 6 protein (*Bcl6*) in T24 and Ramos cells [15]. Previous studies also inferred that miR-127 has a negative effect on clonogenic survival in irradiated human endothelial cells [16]. More importantly, miR-127 is down-regulated in rat hepatocarcinomas induced by a methyl-deficient diet [17]. To further illuminate the roles of miR-127 in livers, the present work sought to elucidate whether and how miR-127 and its possible target genes are involved in LR by focusing on the first 24 h after PH. Our data suggest that the down-regulation of miR-127 might contribute to the proliferation of hepatocytes by targeting SET domain-containing protein 8 (*Setd8*) and *Bcl6* during the first 24 h after PH.

## Results

### MiR-127 Is Significantly Down-regulated and Inversely Correlates with the Expression of Bcl6 and Setd8 during the First 24 h after PH

**Figure 1:**
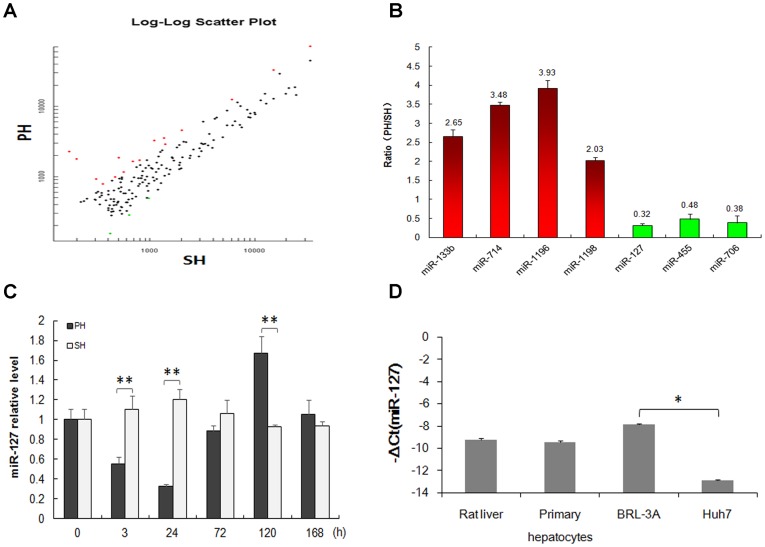
MiR-127 was prominently down-regulated at 24 h after partial hepatectomy (PH) . (**A**) Scatter plot of miRNA expression in PH rats versus SH rats at 3 h post surgery quantified by miRNA microarray which contains 988 human, 627 mouse, and 350 rat miRNA probes. (**B**) Seven differentially expressed rat miRNAs were identified and ratio values >1.5 or <0.7 were considered up-regulated (red) or down-regulated (green) in PH rats compared to SH rats. MiR-127 was identified with at least 3-fold differences between the two groups. (**C**) The relative expression of miR-127 during liver regeneration by qRT-PCR analysis. (**D**) MiR-127 expression levels in rat liver tissues, primary hepatocytes, BRL-3A and Huh7 cells by qRT-PCR analysis. MiR-127 levels were normalized to that of U6. Data from three independent experiments are shown as the means ± SD. (*P<0.05, **P<0.01).

To determine the miRNA expression pattern in the early phase of LR, we performed a comprehensive miRNA expression profiling analysis 3 h after PH or sham operation (SH) (Figure 1A). Among these miRNAs, we identified 4 up-regulated and 3 down-regulated rat miRNAs, in which miR-127 had at least 3-fold difference in expression between PH and SH (Figure 1B). We then examined the expression levels of miR-127 during the whole process of LR by qRT-PCR and found that miR-127 was down-regulated at the early stage and was especially prominent at 24 h after PH, when miR-127 expression displayed an almost 4-fold reduction compared to the SH group. In contrast, miR-127 was up-regulated at the late stage (120 h), in which miR-127 exhibited a 2-fold difference in expression between PH and SH groups (Figure 1C). To further investigate the role of miR-127, we measured the expression levels of miR-127 in rat liver tissues, primary hepatocytes, BRL-3A and Huh7 cells. Results show that the expression levels of miR-127 in rat liver tissues, rat primary hepatocytes and BRL-3A cells were relatively higher than that in human Huh7 hepatocarcinoma cells, and there were no significant difference among rat liver tissues, primary hepatocytes and BRL-3A cells (Figure 1D).

**Figure 2:**
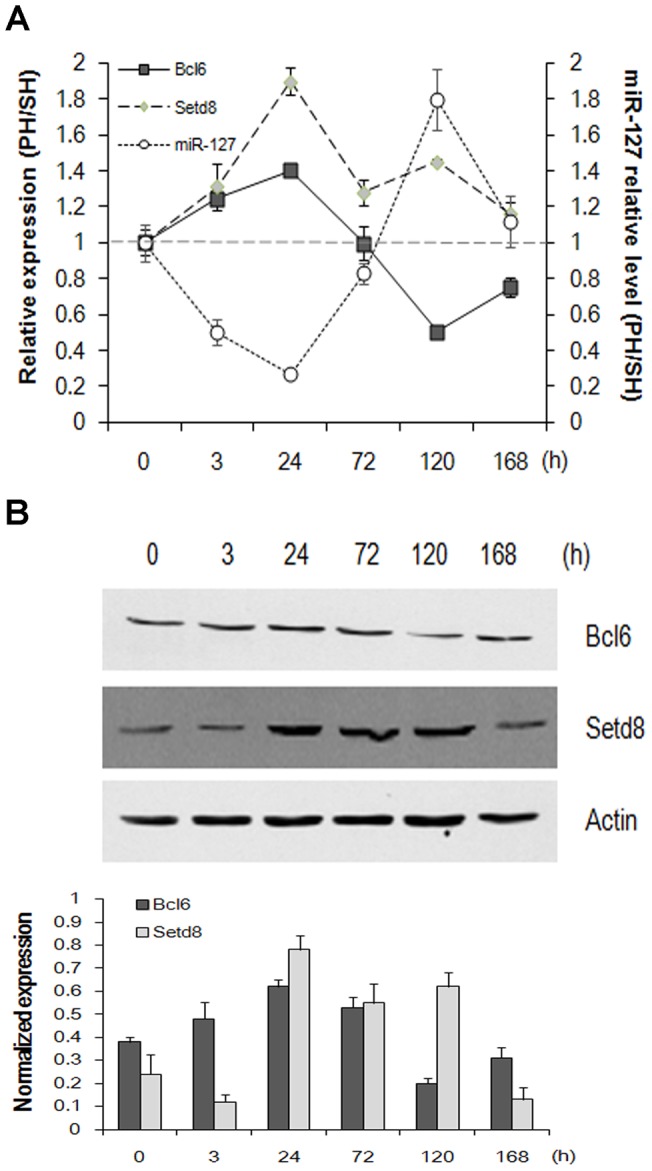
MiR-127 expression inversely correlates with the expression of Bcl6 and Setd8 during liver regeneration. (**A**) The mRNA expression of Bcl6 and Setd8 were analyzed by qRT-PCR. β-actin was used as the internal control. Data from three independent experiments are shown as the means ± SD. (**B**) The protein expression of Bcl6 and Setd8 were analyzed by Western blotting. The quantification of bands of Bcl6 and Setd8 were performed by densitometry. Actin was used as a control. Representative data from one of three independent experiments are shown.

To determine how miR-127 participates in the regulation of LR, we sought to identify miR-127 target genes during LR. Bioinformatic tools for putative miR-127 target genes (TargetScan, miRanda and Pictar algorithms) were used. We selected 24 putative miR-127 target genes that could be involved in regulation of biological or cellular processes as assigned by the pathway classification analysis of these genes (Table S1) (Molecular Annotation System, MAS, http://bioinfo.capitalbio.com/mas). Furthermore, by qRT-PCR and Western blot analysis, we observed a significant up-regulation of Bcl6 and Setd8 on both the mRNA and protein levels in liver tissues 24 h after PH, a time at which miR-127 is maximally decreased (Figure 2, A and B). Over 24 h after PH, the expression of Bcl6 decreased and was maintained at a relatively lower level until the late stage of LR. In contrast, a biphasic induction of the histone H4 monomethylase Setd8 was observed. The first peak occurred at 24 h after PH, and a subsequent peak in expression occurred at 120 h. Setd8 expression returned to almost pre-resection levels by 168 h (Figure 2B). Meanwhile, the SH group showed no significant alteration in expression (data not shown). These data indicated an inverse correlation between the putative target genes *Bcl6*, *Setd8,* and *miR-127* expression on both mRNA and protein levels during the first 24 h after PH.

### MiR-127 Modulates Rat Liver Cell Growth

**Figure 3:**
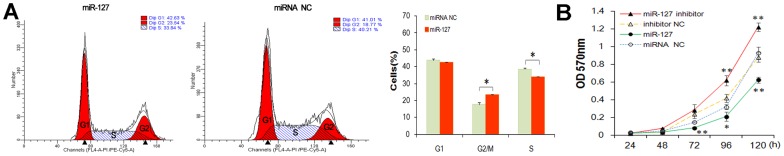
MiR-127 modulates rat liver cell growth. (**A**) Cell cycle of BRL-3A cells transfected with miR-127 mimics (miR-127) or miRNA negative control (miRNA NC) were analyzed by flow cytometry. (**B**) Proliferation of BRL-3A cells transfected with either miR-127 mimics (green), miR-127 inhibitor (red), miRNA NC (blue) or inhibitor NC (yellow) was examined at the indicated time by methylthiazol tetrazolium. Data from three independent experiments are shown as the means ± SD. (*P<0.05, **P<0.01).

To evaluate the effect of miR-127 in regulating rat liver cell growth, BRL-3A immortalized rat liver cells were transfected with miR-127 mimics or a negative control (NC) to up-regulate miR-127 expression levels in liver cells. A cell cycle analysis and a methylthiazol tetrazolium (MTT) cell proliferation assay were employed. As shown in Figure 3A, the percentage of G2/M phase cells in miR-127 and miRNA NC groups was (23.32±0.37)% and (17.65±1.28)%, respectively, and the percentage of S phase cells was (34.00±0.24)% and (38.47±0.84)%, respectively, indicating that a subpopulation of cells is arrested in the G2/M phase by transfection with miR-127 mimics. In addition, miR-127 markedly reduced BRL-3A cell growth at 72 h (*P*<0.01), 96 h (*P*<0.05) and 120 h (*P*<0.01), while miR-127 inhibitor promoted BRL-3A cell growth at 96 h (*P*<0.01) and 120 h (*P*<0.01) compared with the NC groups (Figure 3B) by the MTT cell proliferation analysis. Our results suggest that miR-127 may have a negative regulatory effect on the cell growth of rat liver cells.

### MiR-127 Down-regulates Bcl6 and Setd8 Expression

**Figure 4:**
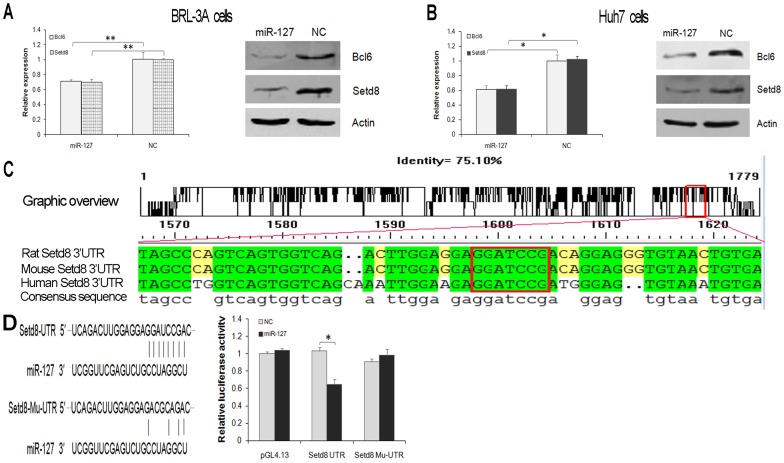
MiR-127 down-regulates Bcl6 and Setd8 expression. (**A** and **B**) BRL-3A (**A**) and Huh7 (**B**) cells transfected with miR-127 mimics or miRNA NC were analyzed by qRT-PCR (left) and Western blotting (right), respectively. Actin was used as a control. (**C**) Graphic overview of the conserved 3′UTR sequences of Setd8 among rat, mouse and human. The “seed sequences” in the 3′UTRs of Setd8 mRNA were indicated (red box). (**D**) The repression of Setd8 gene mediated by the 3′-UTR was analyzed by luciferase reporter assay. BRL-3A cells were cotransfected with a luciferase reporter vector containing the 3′-UTR or the mutated 3′-UTR (Mu-UTR) of Setd8 and miR-127 mimics (miR-127) or the negative control (NC). The pGL4.13 control vector was used as a control. The wild-type and mutated miR-127-binding sites in the 3′-UTR of this gene were indicated (left). Data from three independent experiments are shown as the means ± SD. (*P<0.05, **P<0.01).

To address the regulation of cell growth mediated by miR-127, we examined the expression of the candidate target genes *Bcl6* and *Setd8* in BRL-3A cells after treatment with miR-127 mimics, and found that the expression of these genes were reduced after treatment with miR-127 mimics compared to NCs at both the mRNA and protein levels (Figure 4A). In order to verify whether this down-regulation was only occurred in rat liver cells and explore the potential significance of miR-127 in human liver disease, HCC cell line, Huh7 cells were treated by the same way, and the expression of these target genes was examined. As shown in Figure 4B, treatment with miR-127 mimics caused significant down-regulation of Bcl6 and Setd8 at both the mRNA and protein levels.

To confirm that the inverse relationship of miR-127 expression and that of its predicted target *Setd8* is mediated by the 3′-UTR, we first determined the 3′-end of the full-length *Setd8* transcript by 3′ rapid amplification of cDNA ends (RACE) (GenBank accession JN896763). The 3′-end of the *Setd8* mRNA indeed included a “seed sequence”, and multiple alignments of the sequences of 3′-UTRs revealed higher sequence similarity among rat, mouse and human (Figure 4C). We then fused the 3′-UTR region of *Setd8* to a luciferase reporter to validate the effects of miR-127 mimics on the wild-type and mutant plasmids. As shown in Figure 4D, miR-127 markedly repressed the expression of the luciferase construct that contained the original miR-127 binding site (*Setd8* UTR), but not the mutant binding site (*Setd8* Mu-UTR). These results suggest that Setd8 is a direct target gene of miR-127 in BRL-3A cells.

### Down-regulation of MiR-127 may Participate in Cell Growth Regulation through Bcl6 and Setd8 during LR

**Figure 5:**
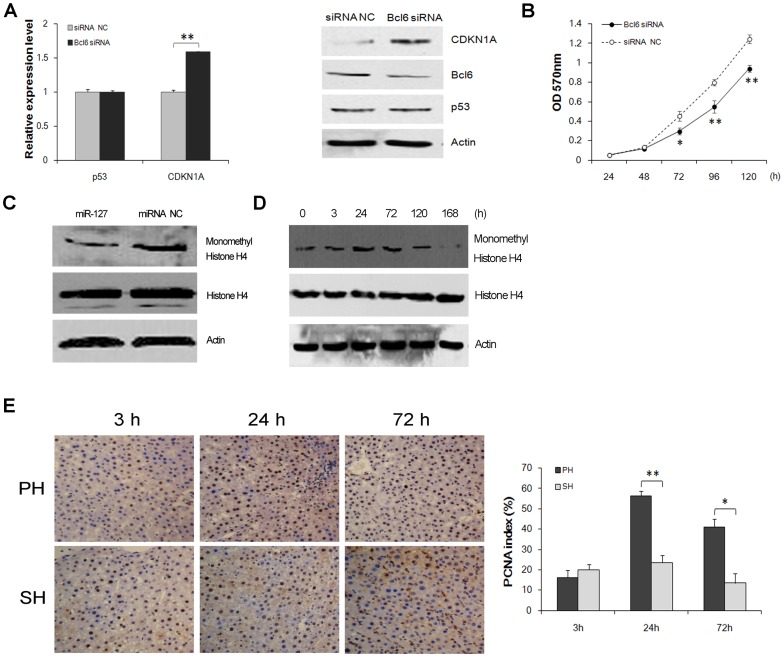
Down-regulation of miR-127 may participate in cell growth regulation through Bcl6 and Setd8 during the first 24 h after PH. (**A**) *CDKN1A*, but not *p*53, is a downstream target gene of Bcl6. BRL-3A cells were transfected with Bcl6 siRNA or a negative control (siRNA NC), and the relative expression levels of mRNA (left) and protein (right) were analyzed by qRT-PCR and Western blotting, respectively. Actin was used as a loading control. (**B**) Down-regulation of Bcl6 suppresses hepatocyte proliferation. BRL-3A cells were transfected with Bcl6 siRNA or siRNA NC, and the relative cell proliferation was examined at the indicated time by methylthiazol tetrazolium. (**C**) MiR-127 decreased the protein expression of monomethyl histone H4. The protein expression of cells treated with miR-127 mimics or miRNA NC was analyzed by Western blotting. Actin was used as a control. (**D**) The expression of monomethyl histone H4 during LR was analyzed by Western blotting. Actin was used as a control. (**E**) Liver sections were stained with PCNA (400× magnification) from 3 to 72 h after PH. All the data were obtained from at least three independent experiments and are shown as the means ± SD. (*P<0.05, **P<0.01).

As a transcriptional repressor, Bcl6 mediates cell survival, proliferation and differentiation by down-regulating over 500 direct target genes, such as *p53*, *ATR*, *CHEK1* and *CDKN1A*, during lymphomagenesis [18], [19], [20]. Among these direct targets, we observed that CDKN1A was markedly induced after transfection with Bcl6 siRNA at both the mRNA and protein levels, but the expression of p53 was not statistically altered (Figure 5A). This result indicated that CDKN1A might be a target of Bcl6, but not p53 in BRL-3A cells. To confirm that the suppression of cell proliferation by miR-127 was mediated by Bcl6, we assessed the effect of Bcl6 siRNA on hepatocyte proliferation. Results show that Bcl6 siRNA strongly repressed BRL-3A cell proliferation at 72 h (*P*<0.05), 96 h (*P*<0.01) and 120 h (*P*<0.01) (Figure 5B). Additionally, because Setd8 has a role in cell cycle regulation by monomethylating histone H4 on Lys20 (H4-K20me1) [21], [22], we measured the expression levels of H4-K20me1 and histone H4 in BRL-3A cells treated with miR-127 mimics or miRNA NC. Results show the transfection of miR-127 mimics notably reduced H4-K20me1 expression but had no effect on histone H4 (Figure 5C). To confirm that the regulatory role of Setd8 during LR was mediated by H4-K20me1, the expression pattern of H4-K20me1 was determined by Western blotting. As shown in Figure 5D, H4-K20me1 was maximally up-regulated at 24 h after PH, and then decreased gradually, its expression was almost below the levels in normal liver tissues at 168 h after PH. These data indicate that miR-127 may regulate the progression of LR via the monomethylating modification of histone H4 mediated by Setd8. Together with the observation that compared with the SH group, the PH group showed an increased proliferative activity at 24 h after liver section (Figure 5E), we speculate that down-regulation of miR-127 may participate in cell growth regulation through Bcl6 and Setd8 during LR.

### MiR-127 Is Down-regulated by Hypermethylation of a CpG Island in the Promoter Region 24 h after PH

**Figure 6:**
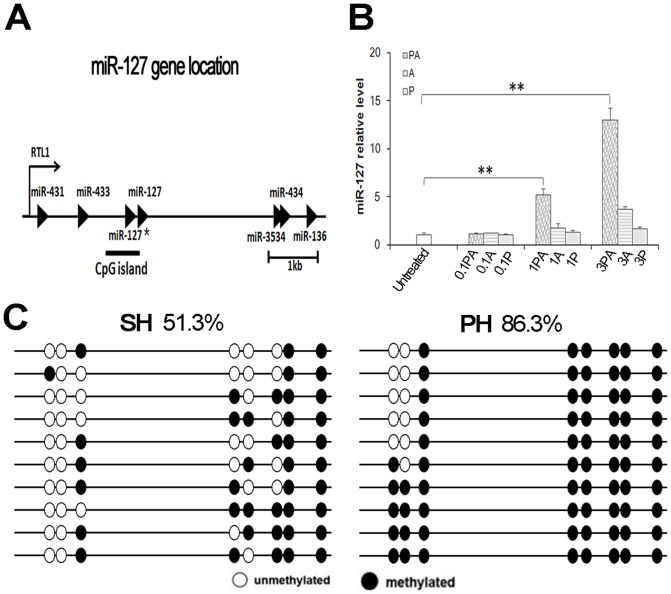
*MiR-127* is induced from its promoter by DNA demethylation and histone deacetylase inhibition. (**A**) Schematic representation of the CpG island on the promoter region of the *miR-127* gene. (**B**) MiR-127 is highly induced by 5-Aza-CdR and PBA treatment. BRL-3A cells were treated with 5-Aza-CdR and/or PBA, and the miR-127 expression level was analyzed by qRT-PCR. U6 RNA expression was used as a loading control. 0.1A, 0.1 μmol/L 5-Aza-CdR; 1A, 1 μmol/L 5-Aza-CdR; 3A, 3 μmol/L 5-Aza-CdR; 0.1P, 0.1 mmol/L PBA; 1P, 1 mmol/L PBA; 3P, 3 mmol/L PBA; 0.1AP, combination of 0.1 μmol/L 5-Aza-CdR and 0.1 mmol/L PBA; 1AP, combination of 1 μmol/L 5-Aza-CdR and 1 mmol/L PBA; 3AP, combination of 3 μmol/L 5-Aza-CdR and 3 mmol/L PBA. Data from three independent experiments are shown as the means ± SD. (**P<0.01). (**C**) The CpG island was hypermethylated 24 h after partial hepatectomy (PH). Genomic DNA was obtained from rat liver tissues 24 h after PH or sham operation (SH), the DNA methylation status was determined by bisulfite genomic sequencing (*P*<0.01, Wilcoxon rank-sum test).

MiR-127 is embedded in a CpG island, as determined by the CpG Island Searcher Program (http://cpgislands.usc.edu/; Takai and Jones, 2002) (Figure 6A). To explore a model of miR-127 regulation in rat liver during LR, we first analyzed miR-127 expression levels in BRL-3A cells treated with 5-aza-2′-deoxycytidine (5-Aza-CdR) and 4-phenylbutyric acid (PBA), which inhibit DNA methylation and histone deacetylase, respectively. As shown in Figure 6B, miR-127 was up-regulated 13-fold after the combination treatment with 5-Aza-CdR and PBA but showed no significant difference with either one of the two drugs alone, indicating that miR-127 can be regulated by the synergistic effects of DNA demethylation and the inhibition of histone deacetylase. To assess whether miR-127 expression is regulated by the methylation of its promoter region during the first 24 h after PH, we measured the DNA methylation levels of the promoter region of *miR-127* by bisulfite sequencing. The results show that the DNA methylation level of the CpG island was increased in the PH group comparison to the SH group (mean of 86.3% versus 51.3%, *P*<0.01, Wilcoxon rank-sum test; Figure 6C). These findings suggest that the down-regulation of miR-127 in rat liver during the first 24 h of LR could be mediated by the DNA hypermethylation of its promoter.

## Discussion

LR after PH offers a unique and robust model to study the initiation, progression, and termination of tissue growth *in vivo* [23]. Although several studies addressed the mechanism by which LR is initiated, the data are insufficient to fully understand the rapid response mechanism of the remnant liver to PH. In particular, little is known about the roles of miRNAs in promoting the growth of the remaining livers. Using a rat model of small size graft liver transplantation and a model of 50% hepatectomy, previous studies have found that most miRNAs were down-regulated in the liver grafts and the remaining livers and suggested that down-regulated miRNAs play a pivotal role in promoting the growth of the grafts and the remaining livers [24]. In the present study, we identified several differentially expressed miRNAs by microarray. Then we confirmed that miR-127 is strongly down-regulated and inversely correlates with the expression of the target genes *Bcl6* and *Setd8* during the first 24 h of LR, while the rebound of miR-127 at 120 h post-PH suggests an inhibitory role of miR-127 in the terminal phase. Our study also found that the artificial down-regulation of miR-127 promotes cell proliferation, while up-regulating miR-127 inhibits proliferation in rat liver cells (Figure 3B), which could be partially attributable to G2/M blockage (Figure 3A). Furthermore, cell growth assays in Huh7 cells also indicate that down-regulation of miR-127 may play a proliferation-promoting role in human hepatocarcinoma cells (Figure S1). Together with the observation that compared with the SH group, the PH group showed an increased proliferative activity at 24 h after liver section (Figure 5E). We proposed that the down-regulation of miR-127 may contribute to the initiation of LR and cell cycle progression during the first 24 h after PH, which is characterised by enhanced proliferation.

To further investigate the specific role of miR-127 during LR, it is very important to identify miR-127 target genes. Although several lines of evidence suggest that transcription factors and cell cycle control kinases have a critical role in initiating and enhancing cell passage from G0 to G1 and in proliferation [25], evidence is still required regarding the complicated regulation of these processes during LR. Therefore, we used a Molecular Annotation System to categorise all of the putative target genes of miR-127 that were predicted by the Targetscan, miRanda and Pictar algorithms. Interestingly, we identified *Bcl6* and *Setd8*, which may participate in cell proliferation and cell cycle control, as direct target genes in rat liver cells. As a proto-oncogene, Bcl6 can be repressed by p53 through a p53 response element (p53RE), and a p53-Bcl6 autoregulatory loop may exist [26]. In our study, we found that an inverse correlation between Bcl6 and miR-127 expression on both mRNA and protein levels during the first 24 h after surgery. Further studies found that CDKN1A was induced when Bcl6 was down-regulated in rat liver cells, suggesting that the miR-127-Bcl6 signaling may regulate the expression of CDKN1A during LR. However, according to previous reports, CDKN1A was highly induced 24h after PH and can be regulated by p53-dependent and -independent pathways in the liver [27]. In addition, the CDKN1A promoter also contains p53 binding sites, and its transcription can be induced by p53 [28], which is highly induced during the prereplicative stage of LR [29], [30]. Therefore, it seems to infer that up-regulation of Bcl6 at 24 h after PH may contribute in part to antagonize the p53-mediated induction of CDKN1A. And an in vivo study of miR-127 in rat LR model will be helpful to elucidate the exact mechanisms that govern the expression of CDKN1A. On the other hand, p53 expression was not altered in BRL-3A cells after repression of Bcl6 (Figure 5A) while it was induced in Huh7 cells (Figure S2). This result suggests that the p53-Bcl6 autoregulatory loop does not function in rat liver cells, which is consistent with previous findings that the p53-Bcl6 loop may exist only in primates because of evolutionary race conservation [26]. Together with the observation that Bcl6 siRNA reduced proliferation in BRL-3A cells (Figure 5B), we presumed that the repression of miR-127 may contribute to the regulation of liver cell proliferation via a Bcl6-mediated antagonism to the p53-mediated induction of CDKN1A during the first 24 h of LR.

Another target of miR-127 that we firstly identified in rat liver cells, Setd8, is a member of a family of histone lysine methyltransferases and can monomethylate histone H4 on Lys20. Setd8 protein levels and histone H4 Lys20 methylation are cell cycle-regulated [21], [22]. The inhibition of Setd8 using shRNA results in the arrest of replication forks, the induction of double-stranded DNA breaks and Chk1-mediated cell-cycle arrest in the S and G2/M phases of the cell cycle [31], [32]. In this study, Setd8 demonstrated a high induction between 24 h and 120 h after PH, while the catalysate of Setd8, H4-K20me1, showed a maximal increase at 24 h and a decrease at the terminal stage of LR (Figure 5D). It may imply that H4-K20me1 at the terminal stage of LR might be further di-methylated and/or tri-methylated by other histone H4 methyltransferases such as SUV420H1, SUV420H2 and NSD2. Considering that Setd8 can also modulate the functions of p53 by the methylation of lysine 382 [33], and p53 has been proposed to be expressed at a higher level in rats receiving PH than in the SH group [30]. We speculate that Setd8 might involve in the p53-mediated transcriptional regulation in the terminal stage of LR. Setd8 up-regulation in the late stage of LR implies that there may exist other regulation models besides miR-127, such as the Ser-29 phosphorylation of Setd8 mediated by the Cdk1/cyclinB complex, which may stabilize Setd8 by inhibiting anaphase-promoting complex (APC)-mediated ubiquitination and proteasome-mediated degradation of Setd8 [34], and it must be further studied. Together with that the H4-K20me1 (Figure 5C) and Setd8 levels (Figure 4A) decreased markedly in the miR-127-treated rat liver cells compared to the control group. We suggest a possible epigenetic mechanism in the regulation of LR that consists of the miR-127-mediated up-regulation of Setd8 during the first 24 h of LR.

*MiR-127* gene is located in the imprinted region Dlk1/Gtl2 and transcribed in an antisense orientation to a retrotransposon-like gene (*Rtl1*) [35], and the expression of miR-127 has been shown to undergo DNA methylation regulation in mouse embryos [36] and human cancer cells [15]. To elucidate the activation mechanism of miR-127 in LR, we analyzed the promoter region of the *miR-127* gene in rats and found a CpG island in it, further studies show that the CpG island was highly methylated 24 h after PH by bisulfite genomic sequencing. These results indicate that the down-regulation of miR-127 after PH may be due to the rapid methylation of the promoter of the *miR-127* gene. This conclusion is further supported by the highly induced expression of miR-127 following treatment with 5-Aza-CdR and PBA, which inhibit DNA methylation and histone deacetylase, respectively. Although a recent study showed that promoter of miR-127 was activated by estrogen related receptor gamma (ERRγ) and inhibited by small heterodimer partner (SHP) [37], further researches will be required to determine whether miR-127 is regulated by ERRγ and SHP in rat LR.

In conclusion, miR-127 is down-regulated within the first 24 h after PH due to the rapid methylation of its promoter, and this event facilitates hepatocyte proliferation by releasing Bcl6 and Setd8. Our findings reveal a miRNA-mediated negative regulation pattern that occurs during the first 24 h of LR and suggest an anti-proliferative role for miR-127 in liver cells.

## Materials and Methods

### Ethics Statement

All animals were cared appropriately according to the Institutional Animal Care Instructions approved by the Ethics Committee for Animals of the Second Military Medical University. All experiments were performed in accordance with the Institutional Animal Care Instructions approved by the Ethics Committee for Animals of the Second Military Medical University (Approval ID: SCXK 2007-0003).

### Animals and Surgery

Adult male Sprague-Dawley rats (180–210 g) were randomly assigned to PH and SH groups (25 animals in each group) and fed standard laboratory chow *ad libitum* with free access to water. After an overnight fast, rats were anaesthetized with sodium pentobarbital (30 mg/kg, intraperitoneally), and underwent PH by removal of 70% of total liver mass (left lateral, left median, and right median lobes) according to the method of Higgins and Anderson [38]. For the sham operation (SH), rats underwent laparotomy and liver manipulation under anaesthesia without tissue removal. Rats were offered food and water *ad libitum* after the operation. At the indicated time points (3, 24, 72, 120 or 168 h after surgery, 5 animals in each sub-group), the animals were euthanized, and the remaining liver tissues were collected. Adult rat primary hepatocytes were isolated and plated as described in detail elsewhere [39].

### Global MiRNA Expression Profiling

Liver samples were obtained at 3 h after surgery, total RNA samples isolated by the Ambion miRNA Isolation Kit were used for analysis of miRNA expression changes after 2/3 PH by CapitalBio Corporation (CapitalBio, Beijing, China). MiRNA expression profiling including labeling, hybridization, scanning, normalization, and data analysis was performed at CapitalBio. Fluoresce-in-labeled miRNAs were used for hybridization on each miRNA microarray chip containing 1320 probes in triplicate, corresponding to 988 human (including 122 predicted miRNAs), 627 mouse, and 350 rat miRNAs found in the miRNA Registry (http://microrna.sanger.ac.uk/sequences/). The differential miRNAs were selected using the program Significance Analysis of Microarrays (version 2.1). The alterations were defined as those with either <0.7- or > 1.5-fold changes and the d value <0.05.

### Cell Line and Transfection

An established rat liver cell line, BRL-3A (ATCC number: CRL-1442) and a human hepatoma cell line, Huh-7, were obtained from the Institute of Biochemistry and Cell Biology, Shanghai, China. Cells were cultured in Dulbecco's modified Eagle's medium (Gibco, BRL) with 10% fetal bovine serum (FBS; Gibco, BRL) and were maintained in a humidified 37°C incubator with an atmosphere of 5% CO_2_. MiR-127 mimics, miR-127 inhibitor, or their relative negative control RNAs, Bcl6 siRNA and its negative control RNA were obtained from GenePharma (Shanghai, China). Transfections were performed using a Lipofectamine 2000 kit (Invitrogen, Carlsbad, CA) according to the manufacturer's instructions. Transfected cells were harvested at 24 or 48 h.

### Reverse Transcription and Quantitative Real-time PCR

Total RNA was isolated from the prepared liver samples and cells using TRIzol reagent (Invitrogen), and cDNA was synthesized following the manufacturer's protocols (MBI Fermentas). qRT-PCR was performed with a standard SYBR-green PCR kit (TOYOBO), and the gene-specific PCR amplification was performed using the Applied Biosystems 7300 Sequence Detection system (Applied Biosystems, USA), the qRT-PCR reactions were performed in triplicate and included no-template controls. Relative gene expression was calculated with the 2^−ΔΔCt^ method after normalization to the expression of β-actin or U6 small nuclear RNA [40]. The primers are listed in Table 1.

**Table 1:** Primers Used in qRT-PCR and Vector Construction.

Primer Names	Sequences (5′ > 3′)
miR-127	AGAGGCGGACGGTGTCGTAGTTGAAGTGAGCCGTCCGCCTCTAGCCAAGC
	GATGTCGGATCCGTCTGAGC
	CGGTGTCGTAGTTGAAGTGAG
U6	GTGCCTGCTTCGGCAGCACATA
	TGGAACGCTTCACGAATTTGCGTGTC
β-Actin	CACCCGCGAGTACAACCTTC
	CCCATACCCACCATCACACC
Human-β-Actin	TGATATCGCCGCGCTCGTCG
	ACCCATGCCCACCATCACGC
Bcl6	TCGAGGTCGTGAGGTTGT
	CGGATAAGAGGCTGGTGG
Human-Bcl6	GAGCCTTTGCCCCCAGCCTG
	GGGGTTGGCCACAGGCATCC
Setd8	ATCACCGATGCCAAGAAGC
	CGTCGATGTCATGCAGTTTG
Human-Setd8	GCAAACTTACGGATTTCTACCCT
	CATTCCTTCTTCCTTCCCACTT
p53	CGGCTCCGACTATACCACTAT
	GGACAGGCACAAACACGAA
Human-p53	GCTTTGAGGTGCGTGTTTGT
	TTGGGCAGTGCTCGCTTAG
CDKN1A	GCAAAGTATGCCGTCGTCT
	CAAAGTTCCACCGTTCTCG
MSP*	TTTTTGAGTTTTAGTAGGTTGG
	ACCAAACRATACTCTCCATATA
Setd8 3′-UTR	GCTCTAGATAACCTGTGATAGCAAGAGTGGG
	GATGGCCGGCCCCACAAACAGGTACAACCAAGAC
Setd8-Mu 3′-UTR	TCAGACTTGGAGGAGACGCAGACAGGAGGGTGTAAC
	GTTACACCCTCCTGTCTGCGTCTCCTCCAAGTCTGA

* Primers used for bisulfite genomic sequencing.

To determine the 3′-end of the full-length *Setd8* transcript, 3′RACE PCR was performed using a FirstChoice RLM-RACE kit (Ambion, USA). The RACE experiments were performed according to the instructions included in the kit. PCR products were cloned into the TA cloning vector pMD18-T (Takara, Dalian, China) and sequenced.

### Cell Proliferation Assay by Methylthiazol Tetrazolium

Cells transfected with miR-127 mimics, miR-127 inhibitor, Bcl6 siRNA or the corresponding NCs in 6-well plates were re-seeded onto 96-well plates at a density of 1000 cells per well 24 h after transfection. At the indicated time points (24, 48, 72, 96, 120 h), 20 μL of a MTT solution (5 mg/mL) was added, and the cells were incubated for 4 h. Subsequently, 150 μL dimethyl sulfoxide (DMSO) was added to each well to dissolve the crystals. The absorbance of each sample was recorded at 570 nm 10 minutes (min) after the addition of DMSO.

### Cell Cycle Analysis by Flow Cytometry

Cells were transfected with miR-127 mimics and the negative control. After an additional incubation of 24 h, the cells were harvested. For the cell cycle analysis, the cells were fixed with 75% ethanol for 24 h and then washed with ice-cold phosphate-buffered saline (PBS) containing 2% FBS. Cells were then spun down, resuspended using RNase-containing (1∶100 in dilution) PBS on ice prior to staining with PI and analyzed using a flow cytometer. Data acquisition and analysis were performed using a FACSort Cytometer (FACS, CA, USA). For each analysis, 1×10^5^ cells were scanned. Each experiment was repeated at least three times.

### Luciferase Reporter Constructs, Site-directed Mutagenesis and Luciferase Reporter Assay

The 3′-UTR of *Setd8*, which contains the miR-127 response element, was cloned into the pGL4.13 luciferase reporter vector (Promega) between the *XbaI* and *FseI* restriction sites using a directional RT-PCR cloning strategy. Mutant 3′-UTR of *Setd8* was synthesized by PCR. The primers are listed in Table 1. The resulting luciferase reporter constructs, pGL-*Setd8*-UTR and pGL-*Set8*-Mu-UTR, which contain the wild-type 3′-UTR and the mutant 3′-UTR of *Setd8*, respectively, were sequenced to ensure accuracy. BRL-3A cells were seeded in a 24-well plate (1×10^5^ per well) and were transfected with 200 ng of the indicated luciferase reporter constructs with miR-127 mimics or miRNA NC. Each sample was cotransfected with 20 ng of the renilla luciferase control vector pGL4.70 (Promega) to monitor transfection efficiency. The pGL4.13 control vector was used as a control. Forty-eight hours later, all protein extracts were analyzed using the dual luciferase reporter assay system (Promega).

### Western Blot Analysis

Tissues and cells were lysed in RIPA lysis buffer (Promega). The lysates were sonicated and centrifuged at 12,000 rpm at 4°C for 10 min. Equal amounts of protein were separated using 10–15% sodium dodecyl sulphate-polyacrylamide gel electrophoresis and were transferred to nitrocellulose membranes (Amersham Pharmacia, UK). For immunodetection, membranes were incubated with specific antibodies (anti-CDKN1A, anti-Bcl6, anti-Setd8, anti-histone H4, anti-monomethyl histone H4, anti-p53 (Santa Cruz Biotechnology), and anti-actin (Sigma)). The immunoblots were developed using horseradish peroxidase (HRP)-coupled anti-mouse or anti-rabbit secondary antibodies (ProteinTech Group) followed by detection with enhanced chemiluminescence (Pierce Biotechnology). Actin was used as a control.

### Immunohistochemical Analysis

The rat liver tissues were collected at the indicated time points (3, 24 and 72 h) after a PH or SH, and fixed with formalin. The fixed tissues were processed for paraffin embedding and serial 5 μm thick sections were prepared. Proliferating cell nuclear antigen (PCNA) analysis was performed using the labelled streptavidin biotin (LSAB) method. The slides were first incubated with an anti-PCNA antibody (Cell Signaling Technology, MA, USA) and then with a biotinylated secondary antibody (ChemMate ENVISION). All stained sections were examined under a light microscope at a ×400 magnification. The PCNA index corresponds to the number of positively stained cells/total number of cells counted in each section.

### 5-Aza-CdR and PBA Treatment

5-Aza-CdR (Sigma-Aldrich, St. Louis, MO) and PBA (Sigma-Aldrich) were dissolved in PBS and DMSO at concentrations of 3 mmol/L and 3 mol/L, respectively. BRL-3A cells were seeded at 2×10^5^ cells per well in 6-well plates 24 h prior to treatment with 5-Aza-CdR (0.1 μmol/L, 1 μmol/L or 3 μmol/L) and/or PBA (0.1 mmol/L, 1 mmol/L or 3 mmol/L). 5-Aza-CdR was removed after 24 h, while PBA was continuously administered by replacing the medium containing PBA every 24 h for 5 days. DMSO-treated cells were used as controls, and each experiment was repeated at least three times.

### DNA Extraction, Bisulfite Treatment and Genomic Sequencing

Genomic DNA was extracted from liver tissues 24 h after PH treatment with the Axygen genomic DNA purification kit (Axygen Biotechnology). Genomic DNA (0.5 μg) was treated with sodium bisulfite with the Zymo EZ DNA Methylation Gold kit (Zymo Research) and then the Bisulfite-converted genomic DNA was amplified with primers (Table 1.) to detect −266 and +36 of the *miR-127* gene by PCR. The PCR products were gel-extracted, subcloned into pMD-18T vectors (Takara), and transformed into *Escherichia coli.* Candidate plasmid clones were sequenced by Invitrogen, Ltd.

### Statistical Analysis

The bisulfite DNA sequencing results were compared using the Wilcoxon rank-sum test. Other results were compared using Student's *t* test, and the data are expressed as the means and standard deviations of at least three independent experiments. All of the *P* values were two-tailed and were obtained with the SPSS 18.0 software package (SPSS, Chicago, IL). A *P* value <0.05 was considered statistically significant.

## Supporting Information

Figure S1 **Down-regulation of miR-127 promotes cell proliferation in Huh7 cells.** (**A**) MiR-127 inhibition increases the percentage of S phase cells and reduces the percentage of G2/M phase cells in Huh7 cells. Cell cycle of Huh7 cells transfected with miR-127 inhibitor or inhibitor NC were analyzed by flow cytometry. (**B**) MiR-127 inhibition promotes Huh7 cell proliferation. Proliferation of Huh7 cells transfected with either miR-127 inhibitor or inhibitor NC was examined at the indicated time by methylthiazol tetrazolium. (**C**) Down-regulation of miR-127 induces the expression of Bcl6 and Setd8. Cells transfected with miR-127 inhibitor or inhibitor NC were analyzed by qRT-PCR (left) and Western blotting (right), respectively. Actin was used as a control. Data from three independent experiments are shown as the means ± SD. (*P<0.05).
(TIF)

Figure S2 **p53 is induced by silencing of Bcl6 in Huh7 cells.** Huh7 cells were transfected with Bcl6 siRNA or a negative control (siRNA NC), and the relative expression levels of mRNA (left) and protein (right) were analyzed by qRT-PCR and Western blotting, respectively. Actin was used as a loading control. (*P<0.05).
(TIF)

Table S1 Candidate miR-127 target genes.
(XLSX)
