# Supplementary figures and images for: Down-Regulation of MiR-127 Facilitates Hepatocyte Proliferation during Rat Liver Regeneration
Source: PLoS One. 2012 Jun 15;7(6):e39151. doi: 10.1371/journal.pone.0039151 (PMC3376093; doi:10.1371/journal.pone.0039151)

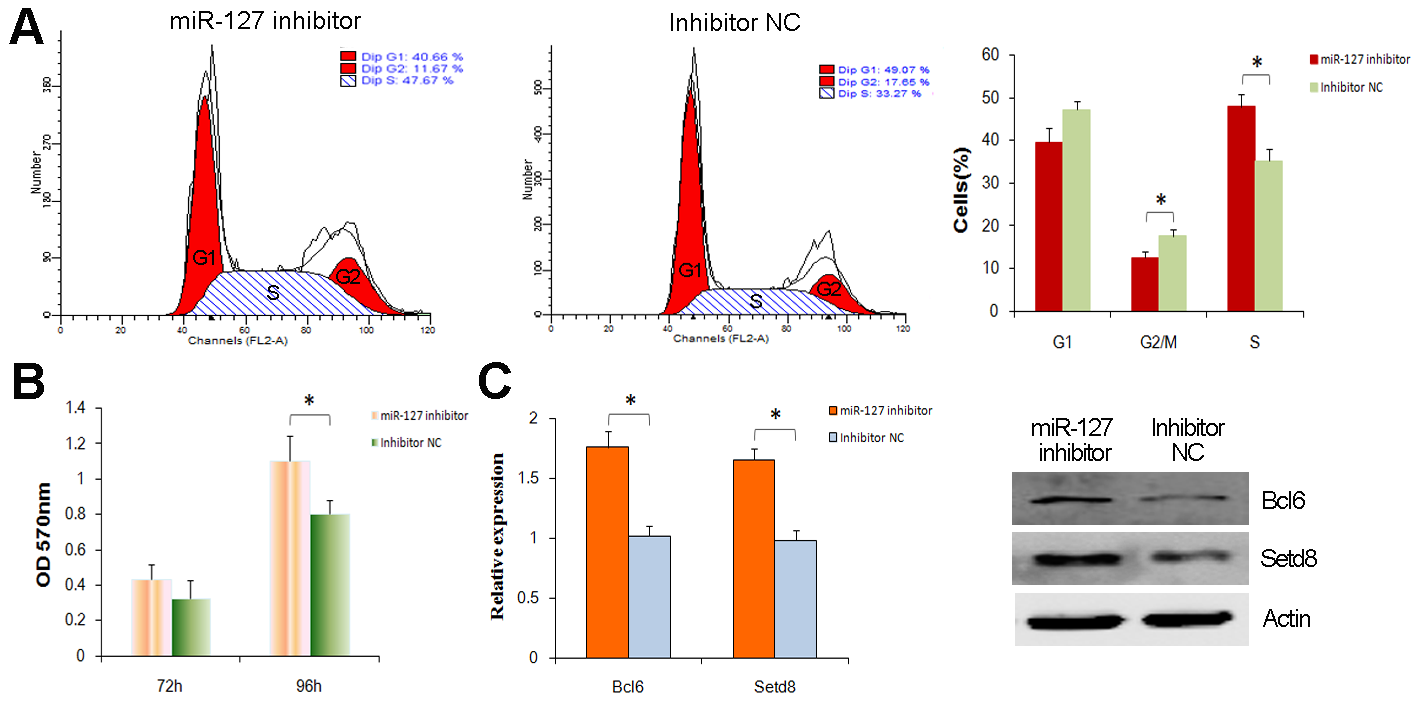

Supplement: Figure S1 — Down-regulation of miR-127 promotes cell proliferation in Huh7 cells. (A) MiR-127 inhibition increases the percentage of S phase cells and reduces the percentage of G2/M phase cells in Huh7 cells. Cell cycle of Huh7 cells transfected with miR-127 inhibitor or inhibitor NC were analyzed by flow cytometry. (B) MiR-127 inhibition promotes Huh7 cell proliferation. Proliferation of Huh7 cells transfected with either miR-127 inhibitor or inhibitor NC was examined at the indicated time by methylthiazol tetrazolium. (C) Down-regulation of miR-127 induces the expression of Bcl6 and Setd8. Cells transfected with miR-127 inhibitor or inhibitor NC were analyzed by qRT-PCR (left) and Western blotting (right), respectively. Actin was used as a control. Data from three independent experiments are shown as the means ± SD. (*P<0.05). (TIF) [file pone.0039151.s001.tif]

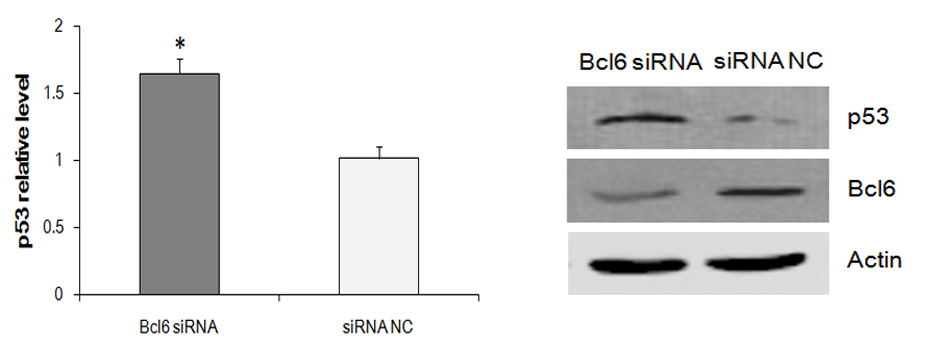

Supplement: Figure S2 — p53 is induced by silencing of Bcl6 in Huh7 cells. Huh7 cells were transfected with Bcl6 siRNA or a negative control (siRNA NC), and the relative expression levels of mRNA (left) and protein (right) were analyzed by qRT-PCR and Western blotting, respectively. Actin was used as a loading control. (*P<0.05). (TIF) [file pone.0039151.s002.tif]
